# Metabolic Profiling Reveals Significant Perturbations of Intracellular Glucose Homeostasis in *Enterovirus*-Infected Cells

**DOI:** 10.3390/metabo10080302

**Published:** 2020-07-23

**Authors:** Zijiao Zou, Jessica Oi-Ling Tsang, Bingpeng Yan, Kenn Ka-Heng Chik, Chris Chun-Yiu Chan, Jianli Cao, Ronghui Liang, Kaiming Tang, Feifei Yin, Zi-Wei Ye, Hin Chu, Jasper Fuk-Woo Chan, Shuofeng Yuan, Kwok-Yung Yuen

**Affiliations:** 1 State Key Laboratory of Emerging Infectious Diseases, Carol Yu Centre for Infection, Department of Microbiology, Li Ka Shing Faculty of Medicine, The University of Hong Kong, Pokfulam, Hong Kong, China; u3006001@connect.hku.hk (Z.Z.); joltsang@connect.hku.hk (J.O.-L.T.); ybp1205@hku.hk (B.Y.); kchik929@connect.hku.hk (K.K.-H.C.); chrisccy@connect.hku.hk (C.C.-Y.C.); caojenny@connect.hku.hk (J.C.); liangrh@hku.hk (R.L.); kmtang20@hku.hk (K.T.); yeziwei@connect.hku.hk (Z.-W.Y.); hinchu@hku.hk (H.C.); kyyuen@hku.hk (K.-Y.Y.); 2 Hainan Medical University-The University of Hong Kong Joint Laboratory of Tropical Infectious Diseases, The University of Hong Kong, Pokfulam, Hong Kong, China; yinfeifeiff@163.com

**Keywords:** *Enterovirus*, metabolic profiling, hNPCs, glucose homeostasis

## Abstract

*Enterovirus* A71 (EV-A71) is a common cause of hand, foot, and mouth disease. Severe EV-A71 infections may be associated with life-threatening neurological complications. However, the pathogenic mechanisms underlying these severe clinical and pathological features remain incompletely understood. Metabolites are known to play critical roles in multiple stages of the replication cycles of viruses. The metabolic reprogramming induced by viral infections is essential for optimal virus replication and may be potential antiviral targets. In this study, we applied targeted metabolomics profiling to investigate the metabolic changes of induced pluripotent human stem cell (iPSC)-derived neural progenitor cells (NPCs) upon EV-A71 infection. A targeted quantitation of polar metabolites identified 14 candidates with altered expression profiles. A pathway enrichment analysis pinpointed glucose metabolic pathways as being highly perturbed upon EV-A71 infection. Gene silencing of one of the key enzymes of glycolysis, 6-phosphofructo-2-kinase (PFKFB3), significantly suppressed EV-A71 replication in vitro. Collectively, we demonstrated the feasibility to manipulate EV-A71-triggered host metabolic reprogramming as a potential anti-EV-A71 strategy.

## 1. Introduction

*Enteroviruses* are non-enveloped, single-stranded, positive sense RNA viruses which belong to the genus *Enterovirus* in the family *Picornaviridae* [1]. The genus *Enterovirus* contains 15 species, including *Enterovirus* A to L and *Rhinovirus* A to C. *Enterovirus* A71 (EV-A71) is one of the major causative agents of hand, foot and mouth disease (HFMD) [2]. Outbreaks of HFMD have been reported worldwide. In mainland China, around 13 million HFMD cases were reported between 2008 and 2015, including at least 123,261 severe cases and 3322 deaths [3]. Severe EV-A71 infections can progress to serious neurological complications, including aseptic meningitis, poliomyelitis-like acute flaccid paralysis, brainstem encephalitis, and non-cardiogenic pulmonary edema [4]. The pathogenesis underlying these clinical features is not fully understood.

Human neural progenitor cells (hNPCs) are stems cells that can differentiate to different lineages of neural cells that are essential for the development and repairing of the nervous system [5]. It has been reported that neurodegenerative diseases may be associated with the dysfunction of neural progenitor cells [6]. Some viruses, such as Japanese encephalitis virus (JEV), Borna disease virus (BDV), and human cytomegalovirus (HCMV), are capable of infecting neural progenitor cells, leading to inhibition or altered differentiation, apoptosis of progenitors, and impaired neurogenesis [7,8,9]. Importantly, viral infections by various DNA and RNA viruses have been shown to induce metabolic reprogramming in host cells to facilitate virus replication [10]. Thus, investigation of the metabolome induced by the neurotropic EV-A71 in hNPCs may provide insights into the pathogenesis of *Enterovirus*-associated neurological complications.

In this study, we used hNPCs as a neural cell-based in vitro model to profile the metabolism of EV-A71 infections. A targeted quantitation of polar metabolites identified 14 candidates with altered expression profiles. A pathway enrichment analysis pinpointed pentose phosphate, gluconeogenesis and glutamate metabolism as the top three pathways affected by EV-A71 infections. Importantly, gene silencing of one of the key enzymes, 6-phosphofructo-2-kinase (PFKFB3), significantly suppressed EV-A71 replication in vitro. Collectively, we demonstrated the feasibility of manipulating EV-A71-induced host metabolic reprogramming as a potential antiviral strategy.

## 2. Results

### 2.1. Establishment of an hNPCs Model for EV-A71 Infection

**Figure 1:**
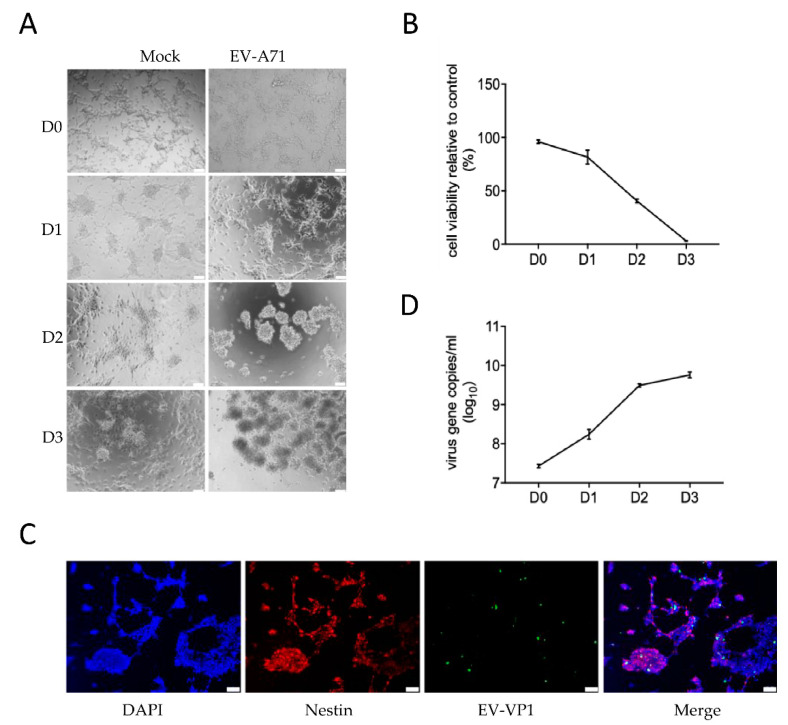
Human neural progenitor cells were susceptible to an *Enterovirus* A71 (EV-A71) infection. The differentiated human neural progenitor cells (hNPCs) in 24-well plates were infected with EV-A71 (multiplicity of infection (MOI) = 1.0). (**A**) Cytopathic effects were recorded under an inverted light microscope daily for three consecutive days. Mock-infected cells were used as negative controls. Scale bar: 50 μm. (**B**) The hNPCs’ viability was determined by thiazolyl blue tetrazolium bromide (MTT) assay daily, from day 0 to day 3 post-EV-A71 infection using the condition as described above. (**C**) EV-VP1 viral protein expression (green) and neural stem cells (red) were detected at day 1 post-infection by fluorescence microscopy. Nuclear DNA was stained by 4′,6-diamidino-2-phenylindole (DAPI, blue). Scale bar: 50 μm. (**D**) EV-A71 genomic copies in the cell culture supernatant were quantified by qRT-PCR at the different time points indicated. The experiments were carried out in triplicate and repeated twice for confirmation. Data are shown as means ± SEM.

To investigate the pathogenesis of EV-A71-associated neurological manifestations, we first exploited hNPCs to establish an in vitro model for the characterization of the metabolic changes induced by an EV-A71 infection. Human neural progenitor cells represent human primary cells that more closely resemble the in vivo physiological state than neuronal cancer cells with abnormal baseline metabolisms. Induced pluripotent stem cells (iPSCs) were cultured and subsequently differentiated into hNPCs. Then, one multiplicity of infection (MOI) of EV-A71 was used for infecting the cells. At 24 h, the virus began to induce cytopathic effects (CPE) in hNPCs, which progressed rapidly and led to extensive cell death after 3 days post-infection (dpi (Figure 1A)). In line with the microscopic observations, hNPCs’ cell viability gradually reduced over time (Figure 1B). These findings suggest that hNPCs were highly susceptible to an EV-A71 infection. Next, immunofluorescent (IF) staining was used to visualize the expression of the neural marker nestin and EV-A71 (viral protein 1) VP1 antigen at 1 dpi. As shown in Figure 1C, the abundant expression (red) of nestin indicated sufficient differentiation of the hNPCs. The expression of the viral VP1 protein (green) was largely co-located with nestin, indicating the neural tropism of EV-A71. Then, to delineate the replication kinetics of EV-A71 in hNPCs, the cell culture supernatant was harvested at 0, 1, 2, and 3 dpi and quantified by reverse transcription—using a quantitative polymerase chain reaction (RT-qPCR) assay. As shown in Figure 1D, the viral load increased by around 1 log unit per day and plateaued at 3 dpi—around a 3-log increase when compared with the baseline at 0 dpi. Taken together, these results demonstrated that hNPCs were highly susceptible to EV-A71 infections and should be a suitable model to investigate EV-A7-induced metabolic reprogramming.

### 2.2. Glucose Metabolism May Play an Important Role in the EV-A71 Life Cycle

**Figure 2:**
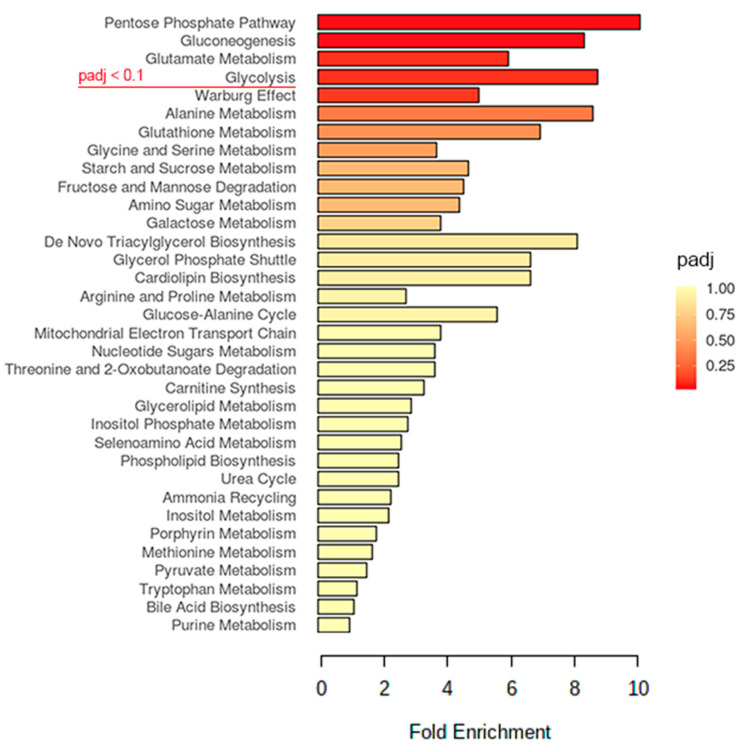
The pathway-associated metabolite sets enrichment analysis of polar metabolites for EV-A71-infected hNPCs. Pathways above the red line achieved padj < 0.1 when compared with the mock-infected control. The analysis was performed on MetaboAnalyst 4.0.

To understand the perturbations of the host metabolism during an EV-A71 infection, we compared the metabolite profiles of hNPCs with or without a virus infection. EV-A71-infected (MOI = 1.0) hNPCs were harvested at 48 h post-infection (hpi) for a targeted metabolomics analysis, followed by a pathway enrichment analysis using the MetaboAnalyst software. A total of 72 polar metabolites in the mass spectrum (MS) features list were first normalized with the total protein of each sample, followed by the quantification of the specific metabolites in EV-A71-infected vs. mock-infected samples (Supplementary Table S1). Subsequently, the statistical significance of the polar metabolites was selected by comparing the groups with/without an EV-A71 infection by using the Student’s *t*-test. After applying the selection criteria of an false discovery rate (FDR) adjusted *p* value (padj) < 0.1 and a fold change threshold of 1.5, 14 significant changed metabolites were determined (Supplementary Table S2) and underwent pathway-associated metabolite sets enrichment analysis based on the Small Molecule Pathway Database (SMPDB) using MetaboAnalyst 4.0 [11]. As shown in Figure 2, phosphate pathways, gluconeogenesis, glutamine metabolism and glycolysis were the top four ranked with padj < 0.1 enriched after EV-A71 infection. It has been reported that the metabolism of glutamate plays an important role in EV-A71 infections [12]. Intriguingly, three of the four top pathways were closely related to glucose’s metabolism. The Warburg effect, ranking below the glycolysis (padj = 0.119), refers to the process of cancer cells and proliferative cell types preferentially utilizing glucose with a high rate of glycolysis to generate pyruvate for lactic acid fermentation in the cytosol [13]. The Warburg effect presents an altered regulation of glycolysis associated with mitochondrial function and influences the pyruvate fate by increasing its provision for anabolic processes [14]. Taken together, the enrichment analysis results suggest that glucose metabolism has a key role in facilitating EV-A71 replication.

### 2.3. Identification of a Metabolic Kinase PFKFB3 Inhibitor with Anti-EV-A71 Activity

**Figure 3:**
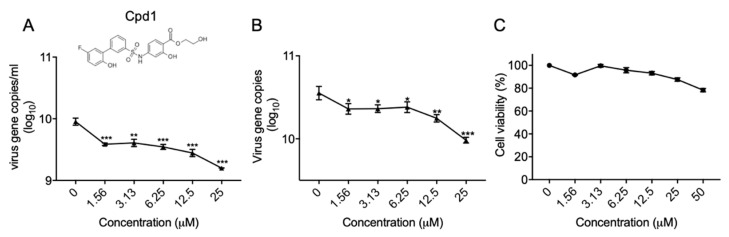
Anti-EV-A71 activity of compound 1 (Cpd1) in hNPCs. The (**A**) cell culture supernatant and (**B**) cell lysate of the virus-infected cells (MOI = 1.0, 48 hpi) were collected for RNA extraction and qRT-PCR detection. (**C**) MTT assay showing the cell viability of hNPCs with different concentrations of Cpd1 treatment for 48 h without virus infection. All the experiments were performed in triplicate. Error bars represent SEM. * denotes *p* < 0.05, ** denotes *p* < 0.01 and *** denotes *p* < 0.001. One-way ANOVA when compared with the 0 µM group (0.05% dimethyl sulfoxide (DMSO)).

To explore the potential of the enriched pathways as therapeutic targets for EV-A71 infections, we purchased commercially available inhibitors of the top four pathways (i.e., padj < 0.1) to determine their inhibitory activity against virus replication. Among the tested compounds, only compound 1 (Cpd1), a potent and selective inhibitor of the metabolic kinase PFKFB3, was shown to inhibit EV-A71 replication while the others were non-effective. As shown in Figure 3, Cpd1 inhibited virus replication in a dose-dependent manner in both the hNPCs’ cell culture supernatant (Figure 3A) and cell lysate (Figure 3B). To exclude the possibility that the observed antiviral activity was caused by Cpd1-induced cell cytotoxicity, the cell viabilities of hNPCs treated with different concentrations of Cpd1 without EV-A71 infection were assessed. Even at the high concentration of 50 µM of Cpd1, the cell viability of hNPCs remained at >80%, indicating that the reduced EV-A71 viral load was due to the inhibition of metabolic kinase PFKFB3 rather than drug-induced cytotoxicity (Figure 3C).

### 2.4. PFKFB3 Is a Druggable Target for Anti-EV-A71 Therapy

**Figure 4:**
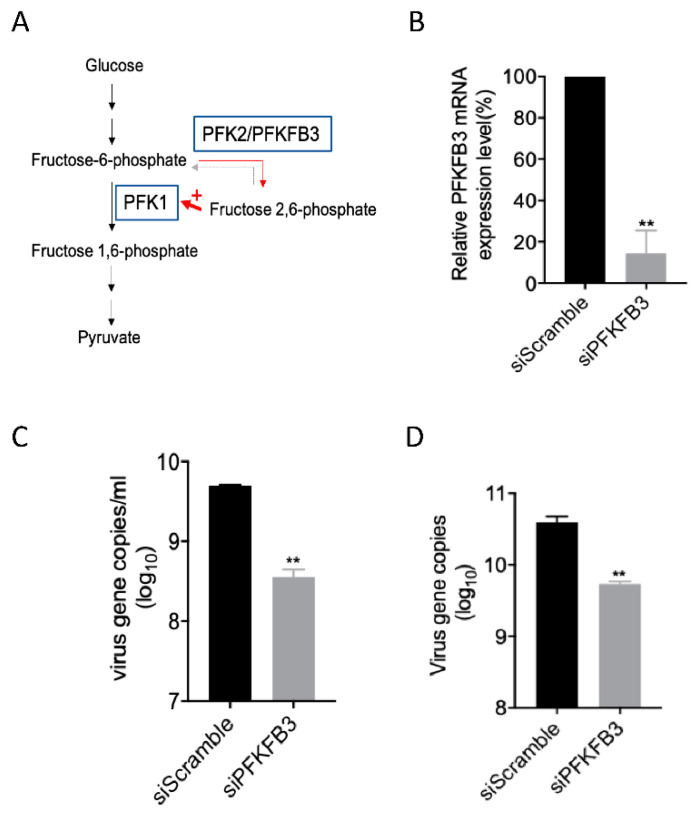
6-phosphofructo-2-kinase (PFKFB3) is crucial for EV-A71 replication. (**A**) Schematic representation of the function of PFKFB3 in the host glucose metabolism. Fructose-2,6-biphosphatase 3, also known as PFKFB3, is a rate-limiting enzyme of glycolysis, which converts fructose-6-phosphate to fructose-2,6-bisphosphate (F2,6 BP). A red + indicates F2,6 BP facilitating the enzyme activity of 6-phosphofructokinase-1 (PFK1) which catalyzes fructose-6-phosphate to fructose-1,6-phosphate. (**B**) 100 nM siPFKFB3 or siScramble was transfected into RD cells for 24 h and then infected with EV-A71 (MOI = 0.05). Supernatant and cell lysates were collected after another 24 h before viral load detection. Knockdown efficiency was evaluated by qRT-PCR assay. PFKFB3 mRNA expression level was reduced when compared to that of the scramble-siRNA treated group. siPFKFB3 knockdown decreased EV-A71 gene copies both in the (**C**) supernatant and (**D**) cell lysates of the infected RD cells. All the experiments were performed in triplicates. Data are shown as means ± SEM. ** denotes *p* < 0.01 when compared with the scramble-siRNA treated group. Student’s *t*-test.

PFKFB3 is a rate-limiting enzyme of glycolysis that converts fructose-6-phosphate to fructose-2,6-bisphosphate (F2,6 BP (Figure 4A)). F2,6 BP is an allosteric activator of 6-phosphofructokinase-1(PFK-1), which is one of the most important regulatory enzymes for stimulating glycolysis. As EV-A71 depends on cellular metabolism to provide energy and to support virus replication, the inhibition of PFKFB3 leading to reduced F2,6 BP might be a potential anti-EV-A71 treatment strategy. To ascertain the role of PFKFB3 in the EV-A71 life cycle, gene silencing with siRNA was performed. As shown in Figure 4B, the siRNA transfection of the EV-A71-susceptible human rhabdomyosarcoma (RD) cells suppressed the PFKFB3 transcript for around 80%. The PFKFB3 knockdown led to around a 1 log reduction of the EV-A71 viral load in both the culture supernatant and cell lysate when compared with scramble-siRNA-transfected RD cells (*p* < 0.01 (Figure 4C,D)).

### 2.5. The PFKFB3 Isoform PFKP Other than PFKM and PFKL Was Responsible for the Reduced EV-A71 Replication

**Figure 5:**
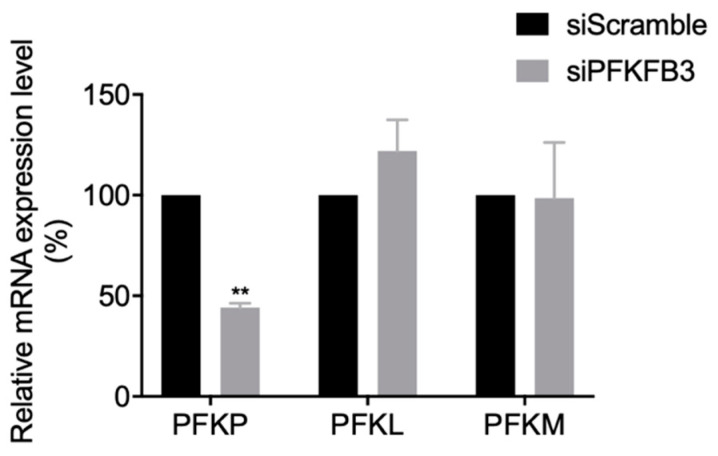
PFKP is responsible for the reduced PFKFB3 expression suppressing EV-A71 replication. The mRNA expression of the three PFK isoform genes (PFKP, PFKL, and PFKM) were analyzed by qRT-PCR after 100 nM siPFKFB3 or siScramble was transfected into rhabdomyosarcoma (RD) cells for 24 h. ** denotes *p* < 0.01 by Student’s *t*-test. The results are shown as the mean ± SEM.

Functioning as one of the most important regulatory enzymes to stimulate glycolysis, PFK has three isoforms including PFKP, PFKM, and PFKL. To pinpoint which of the isoforms affected EV-A71 replication, we examined the mRNA levels of the individual isoforms after PFKFB3 siRNA transfection. When compared with the scramble-siRNA treated control, only PFKP expression was significant reduced (*p* < 0.01), while PFKM and PFKL were not (Figure 5). These results suggested that PFKFB3 may drive the basal glycolytic rates via PFKP activation to facilitate EV-A71 replication.

## 3. Discussion

Viruses are obligate intracellular microorganisms that induce the reprograming of the host cell metabolism to support their own replication. This has been previously reported for various viruses, including HCMV [15], Middle East respiratory syndrome coronavirus (MERS-CoV) [16,17], adenovirus [18], dengue virus, and Zika virus [19]. For example, HCMV-infected cells display an increased dependence on glucose, and upregulate their glycolysis and lactate production. Additionally, glucose depletion is inhibitory to viral replication during HCMV infections [15]. Our recent study on lipogenesis pathways suggested that a MERS-CoV infection activates the sterol regulatory element binding proteins (SREBP) 1 and 2 to enhance the expression of lipogenic enzymes involved in both fatty acid and cholesterol synthesis [16]. Thus, the host metabolism, including the lipid metabolism, has been increasingly investigated as potential antiviral targets in recent years. However, it remains incompletely understood how different viruses induce metabolic reprogramming in host cells.

In this study, we used human primary neural progenitor cells (hNPCs) that are susceptible to EV-A71 infections to comprehensively depict the metabolic profile upon virus infections. Importantly, we demonstrated the key role of PFKFB3, a host kinase with essential roles in glycolysis, in EV-A71 replication and the potential to be an antiviral target. PFKFB (6-phosphofructo-2-kinase/fructose-2,6-bisphosphatase) enzymes are bifunctional enzymes encoded by four different genes (PFKFB1–PFKFB4), which possess different kinetic and distribution properties in their responses to allosteric, hormonal, and growth factors [20]. PFKFB1 encodes the isoforms that were originally identified in liver, muscle, and fetal tissues, while PFKFB2 encodes the isoforms present in the heart and kidney, and some cancer cells [21]. PFKFB3 encodes the isoforms present in the brain, placenta, and adipose tissues, and is the most abundantly expressed PFKFB gene in proliferation and cancer cells [22]. Finally, the PFKFB4 gene encodes the isoforms predominantly found in the testis, although it has also been found in several types of tumor cells [23].

An increased PFKFB3 expression promotes proliferation and carcinogenesis, and its inhibition could be crucial for treating inflammations and cancer [24]. The PFKFB3 expression is induced by endotoxins in human macrophages. Hypoxia enhances the glycolytic flux in macrophages through hypoxia-inducible factor-1 (HIF-1α) and PFKFB3 proportional to the pro-inflammatory activity upregulation in these cells [25]. These in vivo observations suggest that HIF-1α antagonists or PFKFB3 inhibitors might be useful for alleviating inflammatory diseases. Cytosolic viral recognition by secondary interferon signaling has also been demonstrated to upregulate glycolysis in macrophages through the induction of PFKFB3, which promotes the extrinsic antiviral activity of macrophages and forms a crucial component of innate antiviral immunity [26]. Therefore, future studies to delineate the roles of inflammatory responses are warranted to bridge the functionality of PFKFB3 and EV-A71 replication.

## 4. Materials and Methods

### 4.1. Virus, Cell Lines and Chemical Reagents

EV-A71 (Malaysia, gene bank accession number DQ341368.1) was cultured in RD cells in Dulbecco’s Modified Eagle’s Medium (DMEM) supplemented with 2% Fetal Bovine Serum (FBS) as we previously described [27]. The cultured viruses were titrated by a 50% tissue culture infection dose (TCID50) assay. The RD cells (CCL-136) were obtained from ATCC (Manassas, Virginia, USA) and maintained in DMEM supplemented with 10% FBS. Human-induced pluripotent stem cells (iPSCs (IMR90)) were provided by Dr. James Thomson from the University of Wisconsin [28]. The iPSCs were cultured in Matrigel coated 6-well plates with 2 mL of mTeSR^TM^1 medium (STEMCELL). The cell medium was refreshed on a daily basis. The iPSCs were differentiated for 9 days in the differentiation medium consisting of 50% advanced DMEM/F12 medium, 50% neurobasal medium, 1% B27 supplement, 0.5% N2 supplement, 0.5% Glutamax, 10 μg/mL human insulin, 10 μM p-mercaptoethanol and 1% penicillin–streptomycin. All the products included in the differentiation medium were purchased from Gibco (New, Jersey, US). Values of 0.3 μM LDN-193189, 1 μM A83-01, 5 μM CHIR99021, 1 μM RO4929097, 1 μM SU-5402 (Selleckchem, Houston, US) and 0.1 μM of all trans retinoic acid (Sigma-Aldrich, St. Louis, MO, US) were added for hNPC’s induction. Differentiation mediums were changed every two days during the first 4 days and refreshed every single day in the following 5 days.

Alpha-ketoglutaric acid was purchased from Chemcruz (Santa Cruz, US). Benzoic acid was purchased from AccuStandard (New Haven, US). Urea was purchased from USB Corporation (Cleveland, USA). Phospho(enol)pyruvic acid monosodium salt hydrate, 6-phosphogluconic acid trisodium salt, d-ribose 5-phosphate disodium salt hydrate, d-(−)-3-phosphoglyceric acid disodium salt, oxalacetic acid, l (+) lactic acid, fumaric acid, succinic acid, citric acid, malic acid, l-glutamine, l-glutamic acid, l-serine, glycine, d-(+)-glucose, dl-isocitric acid trisodium salt hydrate, sodium pyruvate, dihydroxyacetone phosphate lithium salt, d-fructose 6-phosphate disodium salt hydrate, d-glucose 6-phosphate disodium salt hydrate, adrenaline, dopamine hydrochloride, serotonin hydrochloride, γ-aminobutyric acid, 3,4-dihydroxyphenylacetic acid, homovanillic acid, 5-hydroxyindole acetic acid, glycerol, glycerol 1-phosphate, l-alanine, l-aspartic acid, l-cystine, l-histidine, l-isoleucine, l-leucine, l-lysine, l-methionine, l-phenylalanine, l-proline, l-threonine, l-tyrosine, l-valine, l-asparagine, l-glutamine, l-tryptophan, d-(−)arabinose, fructose, galactose, d-(+)mannose, d-(−)ribose and d-(+)xylose were purchased from Sigma-Aldrich (St. Louis, MO, US). The internal standards including norvaline, glycerol-13C3 and glucose-13C6 were purchased from Sigma-Aldrich too. Water and methanol were purchased from Wako. Methoxylamine hydrochloride and pyridine were purchased from Acros (New, Jersey, US). *N*-methyl-*N*-(trimethylsilyl) trifluoroacetamide with 1% trimethylchlorosilane (MSTFA with 1% TMCS) was purchased from Sigma-Aldrich. All reagents were in HPLC grade equivalent or higher. Ultra-high purity (>99.999%) nitrogen and helium gas were purchased from Linde HKO Ltd (Hong Kong).

### 4.2. Immunofluorescence Microscopy

IF staining was performed according to our previous protocol with some modifications [29]. In brief, hNPCs were seeded on cover slips in the 24-well plates before the EV-A71 infection (MOI = 1). After 24 h, cells were fixed with 4% paraformaldehyde for 15 min at room temperature. Samples were rinsed with cold phosphate-buffered saline (PBS) for three times before they were permeabilized with 0.1% Triton X-100 for 10 min and blocked with 1% Bovine Serum Albumin (BSA) in PBS for 1 h. Samples were then incubated with primary antibodies (anti-EV71-VP1 mouse serum, in house, 1:200 and rabbit-anti-nestin; Sigma-Aldrich, 1:200) at 4 °C overnight, followed by an incubation with a secondary antibody for 1 h at room temperature. Subsequently, samples were mounted with 4′,6-diamidino-2-phenylindole (DAPI (1:1000)) for about 5 min for image capture under an immunofluorescence microscope.

### 4.3. Extraction and Derivatization for GC-MS/MS Analysis

The hNPCs were mock infected or infected with EV-A71 at 1 MOI for 2 days (n = 3 per group). Cells were collected for the extraction of cellular metabolites [30]. Briefly, the cell culture dish (~10^6^ cells) was placed on dry ice and the conditioned medium was aspirated. Cells were washed with ice-cold saline at least 3 to 4 times, which were then suspended in 1 mL of 0.5 mg/L internal standards in 80% ice-cold methanol for 30 min at −80 °C in dry ice. The cells were collected with a cell scraper (less than 30 s). The contents were immediately transferred to pre-chilled cryovials (2 mL). The sample was homogenized after 2 cycles of sonication at 10 microns for 20 s on ice and with a 10 s pause time. The lysate was then centrifuged at 14,000× *g* for 20 min at 4 °C for protein precipitation, and 0.5 mL of the supernatant was dried under a gentle stream of nitrogen at room temperature for derivatization. The dried residue was redissolved and derivatized for 2 h at 37 °C in 40 μL of methoxylamine hydrochloride (30 mg/mL in pyridine) followed by trimethylsilylation for 1 h at 37 °C in 70 μL MSTFA with 1% TMCS. Up to 1 μL of the sample was injected for GC-MS/MS analysis.

### 4.4. Data Acquisition and Statistical Analysis

Metabolome analysis was performed in the Center for PanorOmic Sciences (CPOS), LKS Faculty of Medicine, the University of Hong Kong.

The GC-MS/MS chromatogram was acquired in the SCAN and MRM mode on an Agilent 7890B GC-Agilent 7010 Triple Quadrapole Mass Spectrometer system (Santa Clara, CA, USA). The sample was separated through an Agilent (Santa Clara, CA, USA) DB-5MS capillary column (30 m × 0.25 mm ID, 0.25 μm film thickness) under constant flow at 1 mL·min^−1^. The GC oven program started at 60 °C (hold time 1 min) and was increased from 10 °C min^−1^ to 120 °C, then from 3 °C min^−1^ to 150 °C, and then from 10 °C min^−1^ to 200 °C and finally, from 30 °C min^−1^ to 280 °C (hold 5 min). The inlet temperature and transfer line temperature were 250 °C and 280 °C, respectively. Characteristic quantifier and qualifier transitions were monitored in the MRM mode during the run. Mass spectra from *m*/*z* 50–500 were acquired in the SCAN mode.

The data analysis was performed using the Agilent MassHunter Workstation Quantitative Analysis Software. The linear calibration curves for each analyte were generated by plotting the peak area ratio of the external/internal standard against a standard concentration at different concentration levels. Analytes were confirmed by comparing the retention time and ratio of the characteristic transitions between the sample and standard. The data was then processed to generate a usable csv file including the metabolite names and corresponding concentration for statistical analyses (Supplemental Table S1). MetaboAnalyst 4.0 was used for a univariate analysis and pathway enrichment analysis. For univariate analysis, the statistical significance of the polar metabolites was determined by using the student’s *t*-test. The padj < 0.1 and fold change threshold of 1.5 were used as the criteria for choosing the significant changed metabolites (Supplemental Table S2). Then followed a pathway enrichment analysis of these significantly changed metabolites by using MetaboAnalyst.

### 4.5. Cytotoxicity Assay

Drug cytotoxicity in hNPCs cell was detected by thiazolyl blue tetrazolium bromide (MTT) assay for 48 h post cell–drug incubation according to the manufacturer’s instructions [31].

### 4.6. RNA Extraction and RT-qPCR for Viral Load Detection

Viral load reduction was performed as we previously described [32]. The total cellular RNA was extracted with a Qiagen RNeasy Plus Mini Kit or Thermo Scientific MagJET RNA kit. A qRT-PCR assay was performed using the Roche LightCycler Real-time PCR system. The primers are GAPDH: forward, ATTCCACCCATGGCAAATTC; reverse, CGCTCCTGGAAGATGGTGAT; PanEV: forward, GCCCCTGAATGCGGCTAAT; reverse, ATTGTCACCATAAGCAGCCA; probe: 6FAM-CGGACACCCAAAGTAGTCGGTTCCG-lABkFQ; PFKP: forward, GCATGGGTATCTACGTGGGG; reverse, CTCTGCGATGTTTGAGCCTC; PFKM: forward, GGTGCCCGTGTCTTCTTTGT; reverse, AAGCATCATCGAAACGCTCTC; PFKL: forward, GGAGAAGCTGCGCGAGGTTTAC; reverse, ATTGTGCCAGCATCTTCAGCATGAG.

### 4.7. Antiviral Evaluation after siRNA Knockdown

In the siRNA knockdown experiment, 100 nM of PFKFB3 siRNA or scramble siRNA was mixed with Lipofectamine RNAiMax (Invitrogen, California, US), which was then transfected into RD cells. At 24 h post-transfection, the knockdown RD cells were infected with EV-A71 (MOI = 0.05), at 24 h post-infection, the supernatant and cells were lysed in RLT plus buffer (Qiagen, Venlo, Netherlands) for RNA extraction and viral load detection.

### 4.8. Antiviral Evaluation of Cpd1 in hNPC Cells

The hNPCs were pre-incubated with Cpd1 at a 2-fold diluted concentration or the 0.1% dimethyl sulfoxide (DMSO) control for 2 h, then the cells were infected by EV-A71 (MOI = 1) for 1 h. Next, the cells were washed twice with free DMEM, then replenished with a medium containing different concentrations of the inhibitor for a 48 h incubation. The cell lysates were then collected for RT-qPCR detection as described above.

### 4.9. Statistical Analysis

All the data were analyzed with Graphpad Prism 7 (Graphpad Software Inc, San Diego, US). *p* < 0.05 was considered to be statistically significant.

## 5. Conclusions

Our study has limitations. Besides the later time-point of virus infections (e.g., 2 dpi), an early time-point (i.e., 1 dpi) shall also be performed to fully document the virus-induced metabolomics in the future.

In summary, we demonstrated the feasibility of manipulating EV-A71-triggered host metabolic reprogramming as a potential anti-EV-A71 strategy and pinpointed PFKFB3 as a druggable target for drug development.

## Supplementary Materials

The following are available online at https://www.mdpi.com/2218-1989/10/8/302/s1. Table S1: Polar metabolites that were confirmed in EV-A71 infected and mock infected samples (nmol/mg protein), Table S2: Polar metabolites that were significant changed in EV-A71 infected and mock infected samples.
